# The human *PTGR1* gene expression is controlled by TE-derived Z-DNA forming sequence cooperating with miR-6867-5p

**DOI:** 10.1038/s41598-024-55332-x

**Published:** 2024-02-27

**Authors:** Du Hyeong Lee, Woo Hyeon Bae, Hongseok Ha, Woo Ryung Kim, Eun Gyung Park, Yun Ju Lee, Jung-min Kim, Hae Jin Shin, Heui-Soo Kim

**Affiliations:** 1 Department of Integrated Biological Sciences, Pusan National University, Busan, 46241 Republic of Korea; 2 Institute of Systems Biology, Pusan National University, Busan, 46241 Republic of Korea; 3 Institute of Endemic Diseases, College of Medicine, Seoul National University, Seoul, 03080 Republic of Korea; 4 Department of Biological Sciences, College of Natural Sciences, Pusan National University, Busan, 46241 Republic of Korea

**Keywords:** Z-DNA, Human genome, Transposable element, PTGR1, microRNA, Biochemistry, Computational biology and bioinformatics

## Abstract

Z-DNA, a well-known non-canonical form of DNA involved in gene regulation, is often found in gene promoters. Transposable elements (TEs), which make up 45% of the human genome, can move from one location to another within the genome. TEs play various biological roles in host organisms, and like Z-DNA, can influence transcriptional regulation near promoter regions. MicroRNAs (miRNAs) are a class of small non-coding RNA molecules that play a critical role in the regulation of gene expression. Although TEs can generate Z-DNA and miRNAs can bind to Z-DNA, how these factors affect gene transcription has yet to be elucidated. Here, we identified potential Z-DNA forming sequence (ZFS), including TE-derived ZFS, in the promoter of prostaglandin reductase 1 (*PTGR1*) by data analysis. The transcriptional activity of these ZFS in *PTGR1* was confirmed using dual-luciferase reporter assays. In addition, we discovered a novel ZFS-binding miRNA (miR-6867-5p) that suppressed *PTGR1* expression by targeting to ZFS. In conclusion, these findings suggest that ZFS, including TE-derived ZFS, can regulate *PTGR1* gene expression and that miR-6867-5p can suppress *PTGR1* by interacting with ZFS.

## Introduction

B-DNA, a right-handed double helix, is the most common type of DNA found in organisms. The phosphate backbone of B-DNA is structurally flexible, allowing it to convert to other DNA types within the genome^1^. One of these DNA types is the left-handed double helix form, Z-DNA, discovered using single-crystal X-ray diffraction analysis of double-helical DNA^2^. The bases in B-DNA have an anti-conformation form, whereas Z-DNA has alternating syn-anti conformation. Z-DNA formation is facilitated in sequences that alternate between purine and pyrimidine bases, as purines tend to adopt the syn-conformation more readily than pyrimidines^3^. Although Z-DNA is energetically unstable^4^, it can be stabilized under several conditions, such as negative supercoiling in vivo^5^. During transcription, negative supercoiling occurs behind RNA polymerase, stabilizing Z-DNA^6–8^. Many Z-DNA forming sequence (ZFS) have been identified in actively transcribed gene promoters^9–12^, which could regulate gene expression by altering chromatin conformation^13^. Recent studies have also shown that transposable elements (TEs) can generate multiple Z-DNA regions within the genome^14,15^.

TEs are genetic elements that move from one location to another within a genome and comprise approximately half (45%) of the human genome^16^. TEs are categorized into two classes according to the mechanism by which they move within the genome: retrotransposons (class I) and DNA transposons (class II)^17^. Based on the presence of long terminal repeat (LTR), retrotransposons are sub-divided into LTR retrotransposons and non-LTR retrotransposons, which include long interspersed elements (LINEs) and short interspersed elements (SINEs). While the insertion of TEs into the genome can cause genetic instability, they also regulate various biological functions and contribute to the genetic diversity and evolution of the genome^18,19^. For example, TEs carry regulatory elements, such as promoters, enhancers, and transcription factor (TF) binding sites, which regulate the transcription of adjacent genes^20^. Additionally, TEs can generate transcript variants through alternative splicing events and the introduction of new polyadenylation signal sites^21^.

MicroRNAs (miRNAs) are small non-coding RNA molecules with 19–25 nucleotides that play a variety of biological roles. The miRNA seed region is a short stretch of nucleotides located at positions 2–8 at the 5′ end of the mature miRNA sequence, and it is crucial for determining target specificity^22^. miRNAs usually bind to the 3′ UTR or coding region of mRNA to regulate gene expression post-transcriptionally^23^. Additionally, they can also bind to DNA sequences in the promoter region and be involved in the transcriptional regulation of target genes^24^. Moreover, a recent study reported that miRNA could target ZFS and inhibit the formation of Z-DNA^25^.

Z-DNA can be generated by TEs, and miRNAs can bind to Z-DNA; however, it remains unclear how they regulate transcription of functional genes. In this study, we identified that ZFS, including TE-derived ZFS, could increase prostaglandin reductase 1 (*PTGR1*) expression, suggesting that TEs can regulate gene transcription by generating ZFS. In addition, we found that miR-6867-5p, which can bind to potential ZFS (5′-CACACACA-3′), suppressed the expression level of *PTGR1* by interacting with ZFS.

## Result

### Identification of ZFS and repeat element

The dataset of potential ZFS in the human genome was obtained from Beknazarov’s paper^12^. The paper includes the positions of ZFS predicted by three models: the "Zhunt" model, which uses an algorithm to thermodynamically predict the probability of Z-DNA formation based on the free energy of a given DNA sequence^9^; the "DeepZ" model, which uses a deep learning approach, an artificial intelligence technique that uses existing experimental data for Z-DNA predictions^12^; and Shin's ChIP-Seq model, which identifies Z-DNA sequences using the chromatin immune precipitation with Zaa, consisting of two copies of Zalpha (Za) domains that bind to Z-DNA specifically, followed by next generation sequencing^10^.

**Figure 1:**
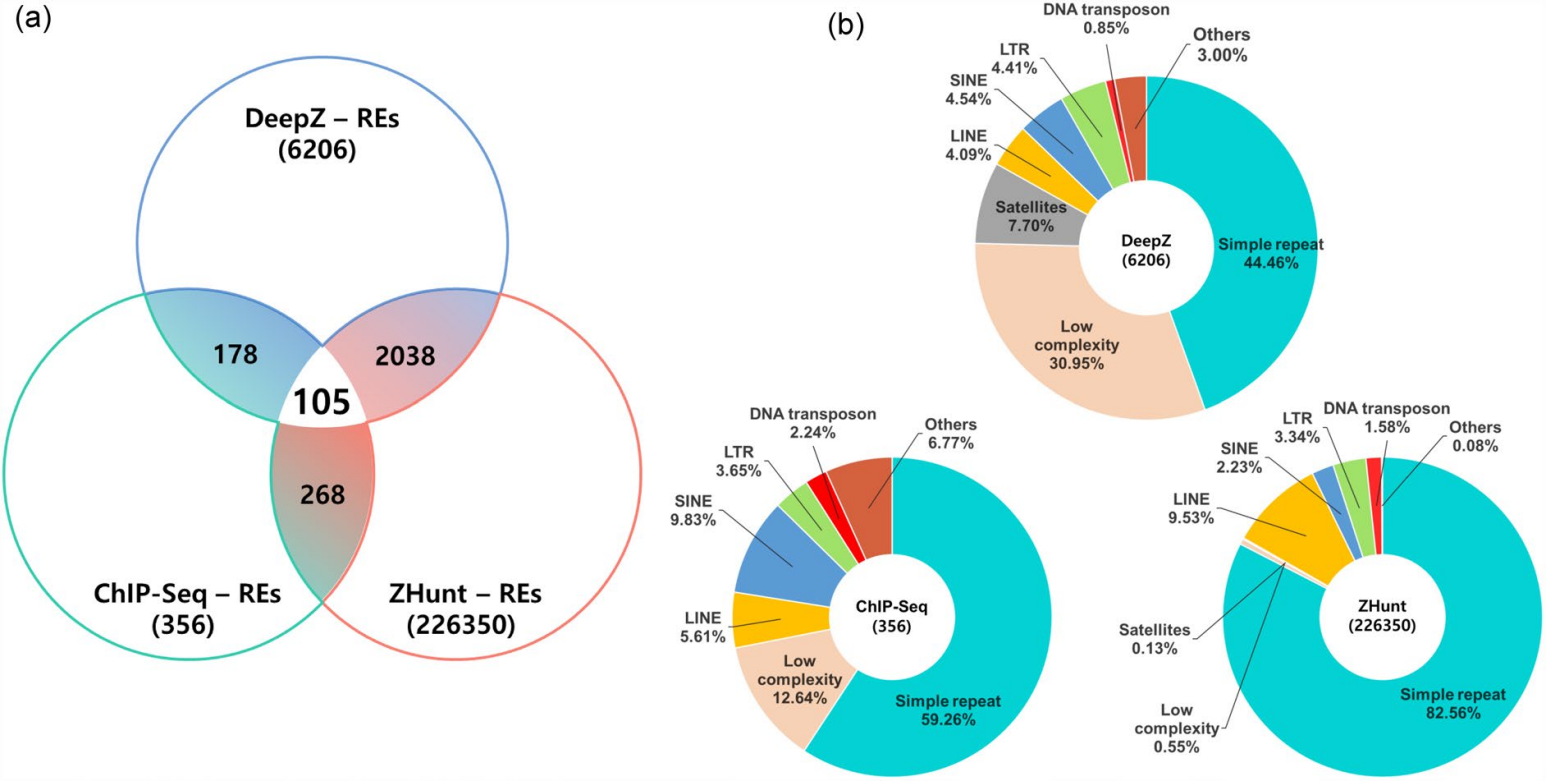
Overlapping regions between ZFS and REs. (**a**) Venn diagram depicting the number of overlapping regions between ZFS and REs in the human genome. Three Z-DNA prediction models (ZHunt, DeepZ, and ChIP-Seq) were used to predict the ZFS, and the RepeatMasker program was used to identify genomic repetitive elements (REs) in the human genome. (**b**) Distribution of RE types overlapping ZFS predicted by three Z-DNA prediction models. RE types were classified into simple repeat, low complexity, satellites, and transposable elements (TEs), including LINE, SINE, LTR, and DNA transposon.

Repetitive elements (REs), including TEs, located in the human genome were identified using the RepeatMasker program. Overlapping regions between ZFS and REs in the human genome were identified using IntersectBed tools (Supplementary Table 1). The number of REs overlapping ZFS predicted by the ZHunt, DeepZ, and ChIP-Seq models was 226,350, 6206, and 356, respectively (Fig. 1a). Among these, there were a total of 105 regions of overlapping REs with ZFS commonly predicted by the three Z-DNA prediction models. Most of the RE types that overlap with ZFS are simple repeats or low complexity, but TEs, such as LINE, SINE, LTR, and DNA transposons, can also be identified (Fig. 1b).

### Overlapping regions of ZFS and TEs in the promoter region

**Figure 2:**
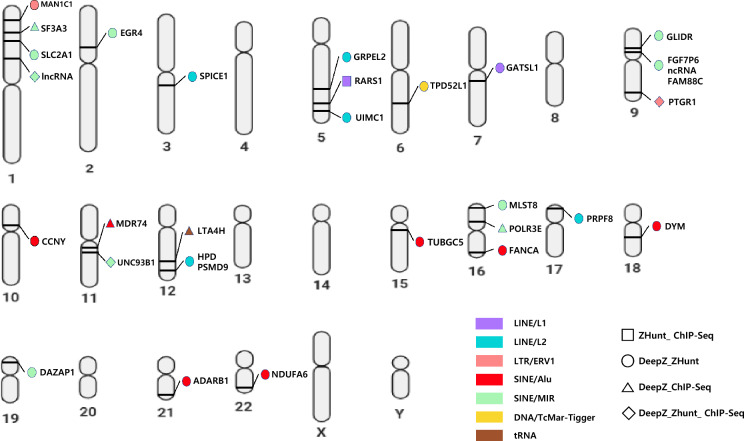
Overlapping regions of ZFS and TEs located in the gene promoter. Overlapping regions located in gene promoters were visualized on the human genome via an idiogram. Each color represents different types of TE (Purple: LINE/L1; Sky blue: LINE/L2; Pink: LTR/ERV1; Red: SINE/Alu; Green: SINE/MIR; Yellow: DNA/TcMar-Tigger; Brown: tRNA). The square represents overlapping TEs and ZFS predicted by ZHunt and ChIP-Seq model; the circle represents overlapping TEs and ZFS predicted by DeepZ and ZHunt models; the triangle represents overlapping TEs and ZFS predicted by DeepZ and ChIP-Seq model; and the diamond represents overlapping TEs and ZFS predicted by all three Z-DNA prediction models.

To confirm the overlapping regions between ZFS and TEs located in the promoter of genes, we only selected those located within 1000 bp upstream and downstream of the transcription start site (TSS) of the genes. The results are displayed on the human genome to visualize the chromosomal locations (Fig. 2). Because potential ZFS were predicted differently depending on the three prediction models, only ZFS predicted by more than two models were selected for a more accurate prediction of Z-DNA regions. As a result, there were 28 overlapping regions, and three TEs overlapping with ZFS that were commonly predicted by all three prediction models were identified in gene promoters: long intergenic non-protein coding RNA (LINC) 01,135 on chromosome 1, prostaglandin reductase 1 (*PTGR1*) on chromosome 9, and Unc-93 homolog B1 (*UNC93B1*) on chromosome 11.

### TE and potential ZFS in *PTGR1* promoter

**Figure 3:**
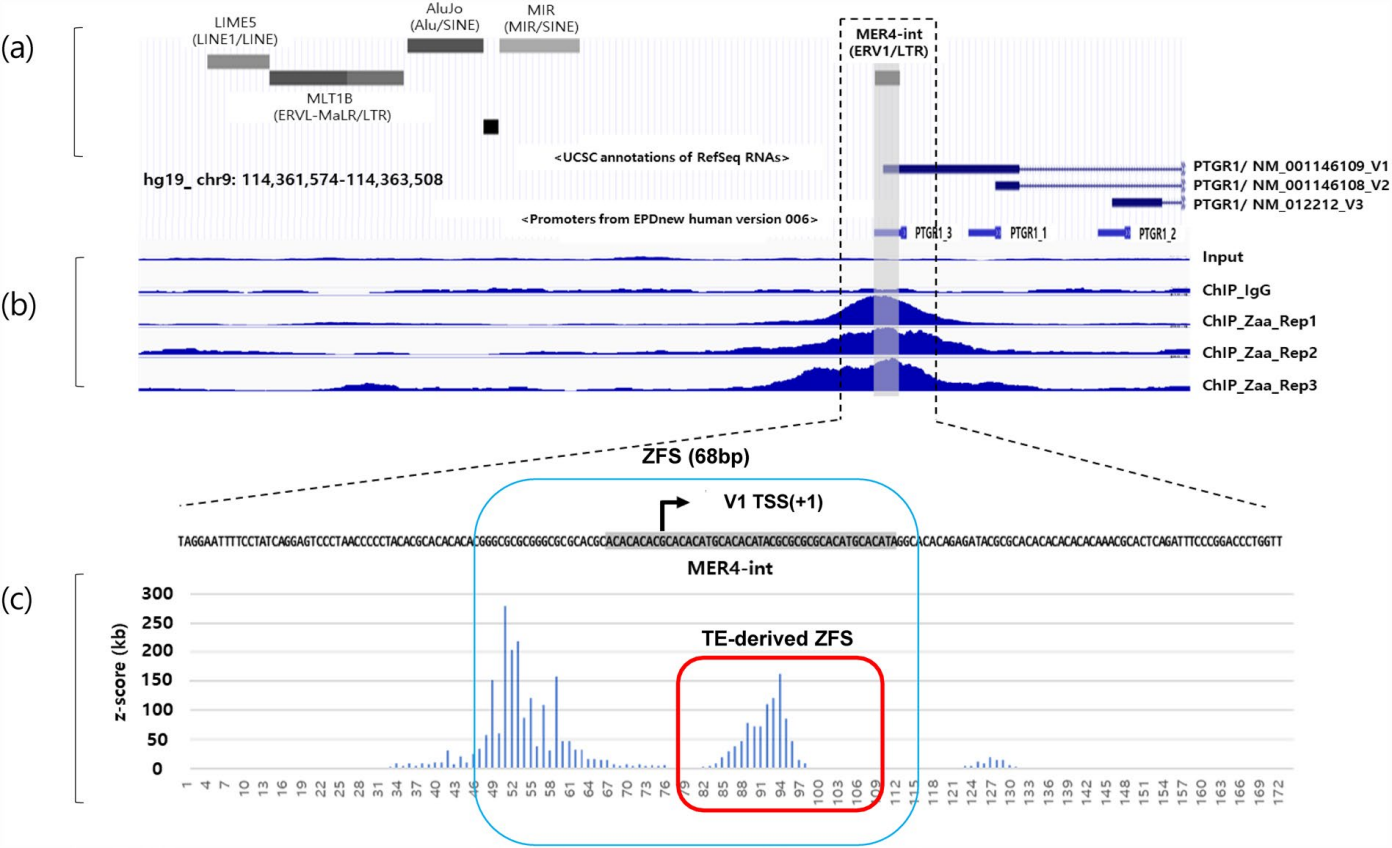
TEs and ZFS in *PTGR1* promoter region. (**a**) Promoter region of the *PTGR1* gene (chr9:114,361,574-114,363,508) is represented on the UCSC genome browser. (**b**) ZFS in the *PTGR1* promoter were predicted using Shin's ChIP-Seq data. The read distributions of input, IgG, and three Zaa replicates were visualized using the UCSC genome browser. The region with high Zaa peaks, marked in blue, indicates ZFS. Input and IgG were used as negative controls. **c** ZHunt analysis was conducted to predict ZFS in the *PTGR1* promoter. The z-score indicates a high probability of adopting the Z-DNA conformation. The shaded gray rectangle represents the MER4-int element.

We focused on the *PTGR1* gene owing to its high z-score and potential ZFS in the TE based oFTn ZHunt analysis. In Zhunt analysis, *PTGR1* had the highest Z-score peak value and was close to TSS. Among the three candidate genes, the average RPKM in 20 tissues was highest for *PTGR1* at 7.32, followed by *UNC93B1* at 0.972 and LINC01135 at 0.188. (Accession: PRJNA280600) TE and ZFS in the *PTGR1* promoter region were analyzed in detail, as shown in Fig. 3. The *PTGR1* promoter region was represented using the UCSC genome browser, and three transcript variants with different TSS were identified: accession no. NM_001146109 (V1), accession no. NM_001146108 (V2), and accession no. NM_012212 (V3) (Fig. 3a). The promoter region contains the medium reiterated frequency repeat4 (MER4)-int, which refers to the internal portion that includes gag, pro, pol, and env genes of endogenous retroviruses (ERV) (Supplementary Fig. 1)^26,27^. According to Shin's ChIP-seq data^10^, high Zaa peaks were observed at the *PTGR1* promoter region (chr9:114,361,949–114,362,333: 384 bp) (Fig. 3b). ZHunt analysis also showed two regions with high z-scores, one within the MER4-int element and the other upstream of the MER4-int element (Fig. 3c). We designated ZFS to include two regions with high z-scores that contained MER4-int and named the ZFS within the MER4-int as TE-derived ZFS.

### Formation of TE-derived ZFS

The entire sequence of MER4-int was obtained using the RepeatMasker program, and the integrated region into the *PTGR1* promoter was confirmed to be the 1116–1160 bp sequence of MER4-int (Supplementary Fig. 2). As shown in Fig. 3, the MER4-int of the *PTGR1* promoter had regions with a high z-score, but no such area was observed in the entire sequence of MER4-int (Supplementary Fig. 3a). To determine how ZFS was generated within the MER4-int, the 1116–1160 bp sequence of the MER4-int and MER4-int inserted into the *PTGR1* promoter were aligned (Supplementary Fig. 3b). Some sequences showed differences in the3 MER4-int inserted into the *PTGR1* promoter compared with the existing MER4-int. These data suggest that the integration of TEs can generate potential ZFS (TE-derived ZFS) through point mutations in the TE sequence.

### Transcriptional activity of the ZFS, including TE-derived ZFS

**Figure 4:**
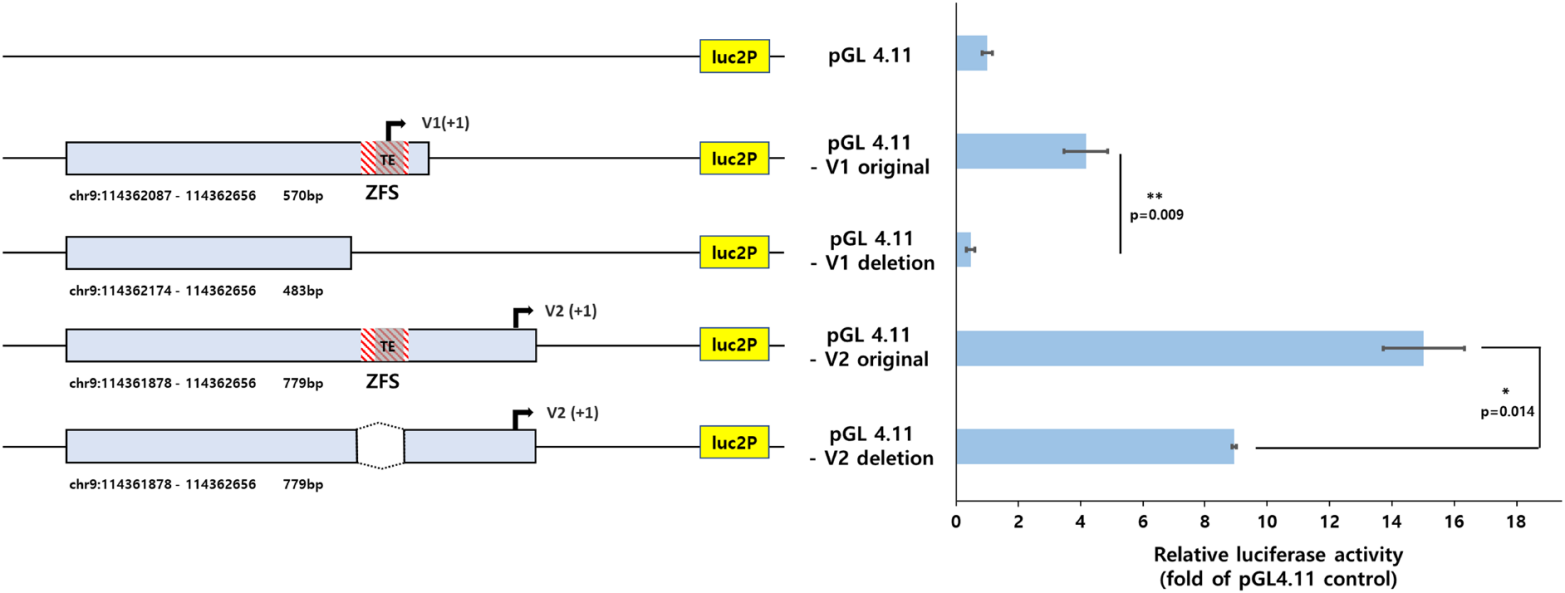
Dual-luciferase reporter gene assays of pGL4.11-*PTGR1* promoter in the HepG2 cell line. (**a**) Schematic representation of pGL4.11-*PTGR1* promoter plasmids. The red-dashed and gray-shaded boxes indicate ZFS and TE (MER4-int), respectively. (**b**) Transcription activities of ZFS, including TE-derived ZFS, in the *PTGR1* promoter. pGL4.11-*PTGR1* promoter plasmids were transiently transfected into HepG2 cell, and luciferase assay was performed 24 h later. The results are expressed as the ratio of the luciferase activity to that of the promoterless-pGL4.11 reporter plasmid. Relative luciferase activity was obtained by normalizing the activity of firefly luciferase with Renilla luciferase. All assays were performed in triplicates, and data were presented with error bar indicating the mean ± standard deviation (SD). Student’s *t* test was used to determine the significance of the results (*p < 0.05, **p < 0.01).

Using a dual-luciferase reporter assay, we examined the regulatory function of the ZFS, including TE-derived ZFS, in the *PTGR1* promoter. We generated four *PTGR1* promoter constructs: the V1 original construct, which includes the TSS of V1 and ZFS; the V1 deletion construct, which lacks both the TSS of V1 and ZFS; the V2 original construct, which includes the TSS of V2; and the V2 deletion construct, in which only the ZFS was removed from the V2 original construct (Fig. 4a, Supplementary Fig. 4). No significant mutations that would change the Z-DNA formation ability were found in each PCR construct generated (Supplementary Fig. 8). After transfecting the plasmids of the pGL4.11-*PTGR1* promoter construct into the HepG2 cells, we measured the promoter activity of each construct (Fig. 4b). A five-fold higher expression was observed in the pGL4.11-V1 original than in the pGL4.11 control vector. The luciferase activity of pGL4.11-V1 original, including ZFS, was significantly increased compared to that of pGL4.11-V1 deletion. Similarly, the deletion of ZFS in the pGL4.11-V2 original resulted in a marked decrease in luciferase expression compared to the pGL4.11-V2 original. These results indicate that the ZFS, including TE-derived ZFS, activates *PTGR1* promoter.

### Interaction between miRNA and ZFS in *PTGR1* promoter

**Figure 5:**
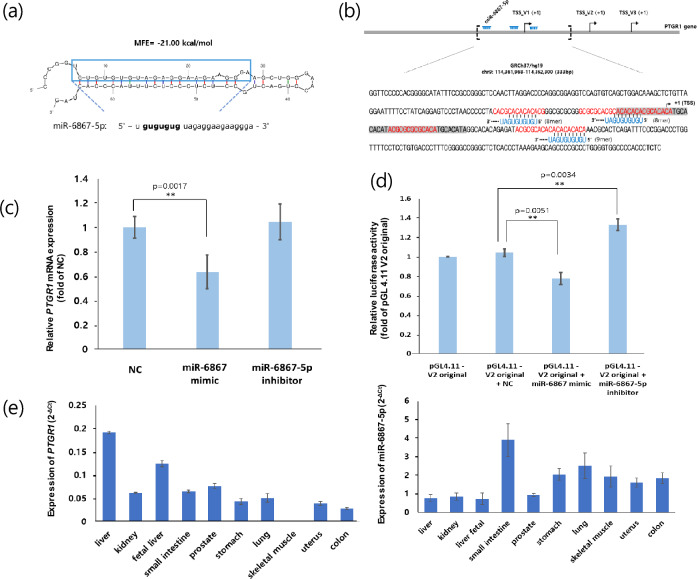
miR-6867-5p suppresses the *PTGR1* gene expression by interacting with ZFS. (**a**) Stem-loop structure of miR-6867 as determined by the mfold web server. The miR-6867-5p sequence and its seed region (5′-GUGUGUG-3′) were obtained from miRbase v22.1, with the seed region in bold. MFE, minimal free energy. (**b**) Target sites of miR-6867-5p in the *PTGR1* promoter. Sequences marked in red and blue indicate the ZFS predicted by ZHunt analysis and the seed region of miR-6867-5p, respectively. The gray-shaded box represents MER4-int. (**c**) HepG2 cells were transfected with miR-NC, miR-6867 mimic, or miR-6867-5p inhibitor, and the *PTGR1* expression level was determined using qRT-PCR (**p < 0.01, Student’s *t* test). (**d**) HepG2 cells were co-transfected with the pGL4.11-V2 original plasmid and miR-NC, miR-6867 mimic, or miR-6867-5p inhibitor. The relative luciferase activity was expressed as the ratio of pGL4.11-V2 original (**p < 0.01, one-way ANOVA). **e** Relative expression of *PTGR1* and miR-6867-5p in human normal sample. All data were presented as the mean ± SD. miRNA, miRNA; TSS, transcription start site; NC, negative control.

To identify the interaction between miRNA and ZFS, miRNAs capable of binding to the *PTGR1* promoter were selected using the miRDB database, among which miR-6867-5p had the highest target score (Table 1). The stem-loop secondary structure of miR-6867 was verified using the mfold web server, and the seed region of miR-6867-5p (5′-GUGUGUG-3') was obtained from miRbase v22.1 (Fig. 5a). We found that the seed region of miR-6867-5p could bind to potential ZFS (5′-CACACACA-3′) predicted by ZHunt analysis in the *PTGR1* promoter region (Fig. 5b).

**Table 1:** miRNA candidates capable of binding to Prostaglandin reductase 1(*PTGR1)* promoter region. Target Score was suggested by miRDB.

Target rank	Target score	MiRNA name	Accession number
1	98	hsa-miR-6867-5p	MIMAT0027634
2	73	hsa-miR-210-5p	MIMAT0026475
3	71	hsa-miR-103a-2-5p	MIMAT0009196
4	71	hsa-miR-103a-1-5p	MIMAT0037306
5	69	hsa-miR-615-5p	MIMAT0004804
6	63	hsa-miR-6732-5p	MIMAT0027365
7	58	hsa-miR-1292-3p	MIMAT0022948
8	55	hsa-miR-4497	MIMAT0019032
9	54	hsa-miR-4666b	MIMAT0022485
10	53	hsa-miR-4783-5p	MIMAT0019946
11	51	hsa-miR-550b-2-5p	MIMAT0022737

To investigate how miR-6867-5p regulates *PTGR1* expression, HepG2 cells were transfected with miR-NC, miR-6867 mimic, and miR-6867-5p inhibitor. HepG2 is a hepatocellular carcinoma cell line with high NRF2 expression, and NRF2 can induce Z-DNA formation by recruiting chromatin remodelers^28^. This makes HepG2 a suitable candidate for testing the Z-DNA transition. (Supplementary Fig. 6).

The results of qRT-PCR analysis showed that miR-6867-5p expression was upregulated in cells transfected with miR-6867 mimic compared with that in cells transfected with miR-NC, while miR-6867-5p levels were downregulated by the miR-6867-5p inhibitor (Supplementary Fig. 5). The *PTGR1* mRNA level was markedly reduced in miR-6867-overexpressed cells (Fig. 5c). A dual-luciferase reporter assay was performed to determine if miR-6867-5p directly targets ZFS in the *PTGR1* promoter. The miR-6867 mimic significantly reduced the luciferase activity of pGL4.11-V2 original, whereas the miR-6867-5p inhibitor increased its luciferase activity (Fig. 5d).

Additionally, the expression pattern of miR-6867-5p and *PTGR1* was examined in various human normal tissues (Fig. 5e,f). The expression of *PTGR1* was the highest in the liver, whereas the expression of miR-6867-5p was relatively low compared with that in other tissues. In addition, *PTGR1* and miR-6867-5p showed an inverse tendency expression in other tissues. Taken together, these results suggest that miR-6867-5p could inhibit *PTGR1* expression by binding to ZFS in the *PTGR1* promoter in human liver.

## Discussion

In this study, overlapping regions between ZFS and TEs were identified in the human genome through data analyses. Among them, overlapping regions located in the promoter of functional genes were selected. As a result, TE-derived ZFS in the *PTGR1* promoter region was found, and ZFS, including TE-derived ZFS, affected transcriptional regulation. Furthermore, the interaction between miRNA and ZFS was investigated, and it was found that miR-6867-5p could bind to ZFS to regulate *PTGR1* expression.

Using a deep learning approach, ZFS was predicted for genomic repeats, which revealed that Z-DNA primarily exists in simple repeats, low complexity regions, and satellite DNA. ZFS included in TEs also accounted for approximately 1–3%^12^. The results of data analysis also showed that a large proportion of ZFS overlapped with a simple repeat and low complexity; moreover, TEs overlapping with ZFS in the human genome were also identified (Fig. 1). Further, detailed information about where the overlapping regions were located in the human chromosome was also obtained (Supplementary Table 1).

Given that TE and Z-DNA could be located in the gene promoter and regulate gene expression, we hypothesized that there would be overlapping ZFS and TEs in the promoter region, which regulates gene expression. Z-DNA formation depends on several factors, including the sequence, Z-DNA binding proteins (ZBPs), chromatin structure, and environmental conditions^12,29^. Especially, the transcription factor NRF2 can induce the binding of a chromatin remodeler, thereby promoting the formation of Z-DNA^30^. On the other hand, for sequences recognized as most crucial in Z-DNA formation, the analysis results revealed that introducing one to two point mutations in the sequence did not significantly alter the likelihood of Z-DNA formation. (Supplementary Fig. 7) thus, ZFS is predicted differently in the human genome according to prediction models. We assumed that ZFS predicted in common by two predictive models and ChIP-seq data would have a higher probability of Z-DNA formation than those predicted by only one model. Therefore, overlapping TEs and ZFS predicted by more than two prediction models were identified in the promoter of genes (Fig. 2). The predicted sequence exhibited the highest affinity of Zaa proteins, which can specifically bind to Z-DNA according to the ChIP-seq results. Additionally, it showed the highest probability of thermodynamic Z transition as calculated by the program^9,10^.

**Figure 6:**
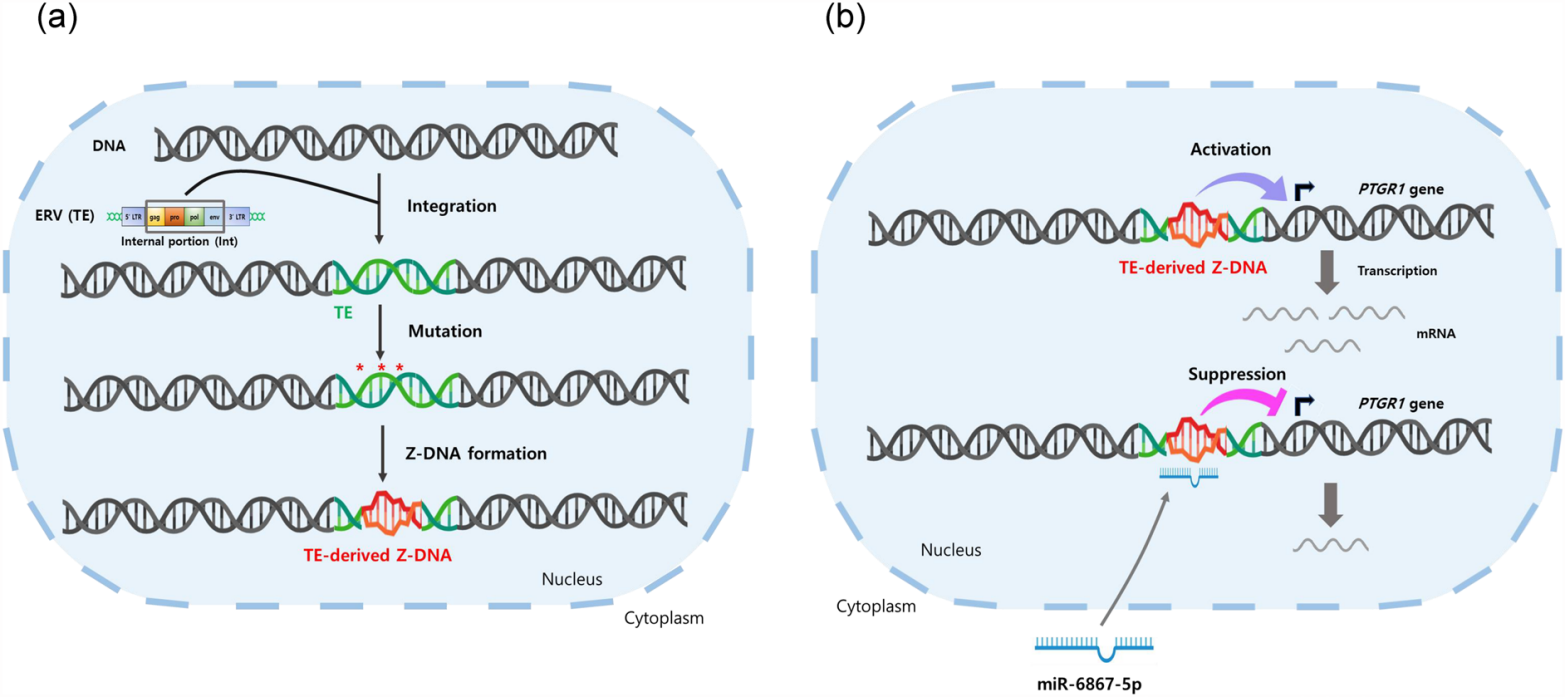
Schematic illustration of *PTGR1* transcriptional regulation. (**a**) Formation of TE-derived ZFS. Mutations can occur within TEs inserted into the human genome, which may result in the formation of Z-DNA. (**b**) The *PTGR1* gene regulation mechanism. ZFS, including TE-derived ZFS, located in the *PTGR1* promoter could facilitate transcription, while miR-6867-5p targets ZFS to suppress its expression.

Several studies have reported that TE could generate Z-DNA. For example, ZFS was found in the Alu retrotransposon of the African green monkey genome^31^, and the signal recognition particle binding site of the Alu retrotransposon overlapped with ZFS^15^. In addition, potential ZFS in the LTR element serving as an alternative promoter was recently confirmed^14^. These previous studies showed that TEs are capable of generating Z-DNA. Likewise, our data showed potential ZFS within the MER4-int sequence in the *PTGR1* promoter (Fig. 3). In particular, mutations in TEs could generate potential ZFS (Fig. 6A). Taken together, TEs have the potential to be ZFS themselves, and they could also acquire the ability to generate Z-DNA through mutations in specific sequences.

Although the function of TEs in the genome is still not fully understood, they have several biological roles in organisms^32^. In particular, TEs could affect the expression of nearby genes by acting as regulatory elements or altering the structure of chromatin^33–35^. For instance, TEs could contain regulatory sequences, such as promoters or enhancers^20^. When TEs are inserted into the promoter of functional genes, they can disrupt and create new regulatory sequences that affect gene expression^36^. In the present study, we found that TE-derived ZFS in the *PTGR1* promoter (Fig. 3) and the ZFS region, including TE-derived ZFS, upregulated *PTGR1* transcription (Fig. 4). Our findings suggest that TEs inserted into gene promoters could regulate gene expression by forming Z-DNA. However, additional research is needed to determine whether the Z-DNA structure is formed in the *PTGR1* gene promoter.

There have been no reports on whether Z-DNA can regulate the expression of *PTGR1*. In this study, we showed that ZFS in the *PTGR1* promoter could regulate transcription. The human *PTGR1* gene, an NADPH-dependent alkenal/one oxidoreductase, is involved in the arachidonic acid metabolism pathway and inactivates some eicosanoids^37^. The *PTGR1* gene regulates a variety of physiological and pathological processes, including inflammation^38^ and antioxidant respons^39^. The dysregulation of *PTGR1* is associated with several cancers or diseases^37,40^. *PTGR1* is overexpressed in various cancer cell lines, promoting cancer cell proliferation^41–43^ and increasing resistance to cancer oxidative stress^37,44^. Growing evidence has emphasized that this gene can be used as an effective therapeutic target for various diseases, including cancer^37,40^. Therefore, additional studies on the *PTGR1* regulatory mechanism are needed. *PTGR1* expression is regulated by nuclear factor erythroid 2-related factor 2 (NRF2), a type of TF, and inhibiting NRF2 reduces *PTGR1* expression^44–46^. The relationship between NRF2 and Z-DNA was also described by Zhang et al^30^. The Heme Oxygenase 1 (HO-1) gene promoter region contains ZFS and NRF2 binding sites. NRF2 recruits the BRG1 chromatin remodeling enzyme, which remodels the nucleosome and promotes Z-DNA formation. This formation of Z-DNA facilitates chromatin opening and RNA Pol II recruitment. In addition, BRG1 and Z-DNA formation is necessary for regulating chromatin remodeling and Colony Stimulating Factor 1 (*CSF1*) gene activation^47^. These findings suggest that NRF2 and a chromatin remodeling complex induce Z-DNA formation in the *PTGR1* promoter and that this Z-DNA is necessary for *PTGR1* expression. However, further studies are needed to clarify the association between NRF2, chromatin remodeling enzymes, and Z-DNA in *PTGR1* transcriptional regulation.

Several factors, such as TFs, chromatin remodelers, and ZBPs, can stabilize Z-DNA. In contrast, not many factors have been identified that can inhibit Z-DNA formation. Recent studies have shown that miRNAs can enter the nucleus and bind to DNA in a complementary, thereby regulating gene expression^48,49^. Additionally, miR-324-3p and miR-744 were found to bind to potential ZFS in the promoter region of *RELA* and *ARHGAP5*, respectively, suggesting that miRNAs can inhibit Z-DNA formation^25^. Therefore, we identified miRNAs that could bind complementary to that region. We found that miR-6867-5p binds to ZFS in the *PTGR1* promoter. Because Z-DNA was well-formed in alternating purine-pyrimidine dinucleotide repeat sequences in the order (GC)n > (CA)n > (TA)n^8,50^, miR-6867-5p, with the seed region of GTGTGT, can bind to ZFS regions with (CA)n sequences. miR-6867-5p also decreased *PTGR1* expression, suggesting that miRNAs binding to ZFS could suppress gene transcription by inhibiting Z-DNA formation. In the experiment, not only did luciferase reporter expression decrease, but there was also a reduction in *PTGR1* expression at the RNA level throughout the entire cell. This result contradicts previous studies that demonstrate the interaction between miRNA and double-strand DNA generally enhances gene expression^51^. This suggests that a different structural transition, rather than the general DNA-miRNA triplex interaction, had an additional effect on gene expression. Previous studies have demonstrated miRNA's ability to inhibit Z-DNA transition^25^. Considering the association between Z-DNA transition and gene expression regulation, it is assumed that this results from miRNA inhibiting Z-DNA transition. This suggests that miR-6867 was imported into the nucleus and played a role in regulating gene expression. The results presented in this study confirmed that miRNAs bind to potential ZFS to inhibit gene expression. However, further research is needed to determine the impact of miRNAs binding to ZFS on actual Z-DNA formation.

In conclusion, we demonstrated that ZFS, including TE-derived ZFS, activated *PTGR1* transcription regulation and that ZFS-binding miR-6867-5p suppressed its expression. This is the first report emphasizing that TE could regulate gene transcription by generating ZFSs. In this study, we also discovered a new ZFS-binding miRNA (miR-6867-5p) and confirmed that this miRNA effectively suppresses gene expression (Fig. 6B). These findings provide new insights into the potential of ZFS-binding miRNAs as therapeutic agents for treating diseases associated with *PTGR1* and Z-DNA.

## Methods

### Data analysis

To identify ZFS in the human genome, we used a public dataset (https://github.com/Nazar1997/DeepZ/tree/master/annotation, accessed Dec 16, 2021) by Beknazarov^12^. The chromosomal location of repetitive elements, including TEs, was obtained from the "Repeats" group (RepeatMasker) on the table browser tap in the UCSC genome browser (https://genome.ucsc.edu/) based on GRCh37/hg19 assembly^52^. Subsequently, we identified the overlapping regions of ZFS and REs using the IntersectBed module from Bedtools 2. 29. 1 (https://bedtools.readthedocs.io/en/latest/index.html), and the output files were converted using in house Python codes. The PhenoGram Plot (http://visualization.ritchielab.org/phenograms/plot) was used to show the overlapping regions of ZFS and TEs in the promoter region on the human chromosome. To identify ZFS in the *PTGR1* promoter, we used the ZHunt program and Shin's ChIP-seq data^10^. We rearranged Shin’s data from the hg18 reference genome to the hg19 reference genome using Model-based Analysis of ChIP-Seq (MACS) and then used Integrative Genomics Viewer (IGV) software to display potential ZFS in genomic data.

### Bioinformatics tools

RepeatMasker 4.0.7 (https://www.repeatmasker.org/) was used to analyze repeat sequences in the *PTGR1* promoter obtained using the UCSC Genome Browser. For sequence alignment, the BioEdit program (http://www.mbio.ncsu.edu/BioEdit/bioedit.html) and MEGA7 software (https://www.megasoftware.net/) were used. The Primer3 v0.4.0 program (https://bioinfo.ut.ee/primer3-0.4.0/) was used to design the primers amplifying the *PTGR1* promoter region. miRNA candidates bound to the *PTGR1* promoter were selected via miRDB (https://mirdb.org/). The stem-loop structure of miR-6867 was predicted using Mfold software (http://www.unafold.org/mfold/applications/rna-folding-form.php), and the miR-6867-5p sequence and its seed region were identified using miRbase v22.1 (http://www.mirbase.org/).

### Genomic DNA extraction and PCR amplification

Genomic DNA (gDNA) was extracted from HepG2 using DNeasy Blood & Tissue Kit (Qiagen, Germany), according to the manufacturer’s instructions. DNA samples were quantitated at 500 ng/µl using the ND-1000 UV–Vis spectrophotometer (NanoDrop, USA). This gDNA was used for PCR amplification with the 2 × TOP simple DyeMix (aliquot)-HOT premix (Enzynomics, Republic of Korea). Each of the three constructs, including the promoter region of *PTGR1*, was generated by PCR amplification using one sense (S) primer and three anti-sense (AS) primers: S primer (5′-CTG AGA CCA CCT CTC CTT GC-3′), V1 deletion_AS1 primer (5′-GTG TGC GTG TAG GGG GTT AG-3′), V1 original_AS2 primer (5′-CGC GTA TCT CTG TGT GCC TA-3′), and V2 original_AS3 primer (5′-CTT ACA GGA GCC CGA AGG TT-3′) (Supplementary Fig. 4). The PCR conditions were as follows: initialization at 95 °C for 5 min, 40 thermal cycles of 94 °C for 40 s, primer-specific annealing at 60.5 °C for 40 s, 72 °C for 1 min, and a final elongation step at 72 °C for 5 min. All PCR products were purified with the Exprep Plasmid SV mini (GeneAll, Republic of Korea), Sanger sequencing was conducted by Macrogen (Seoul, Republic of Korea) using the four forward primers and four reverse primers listed above.

### Formation of deletion mutant construct and gene cloning

To create the V2 deletion plasmids, the pGL4.11 vector cloned with the V2 original construct was amplified by PCR using 5′-phosphorylated primers; S, 5′-GGC ACA CAG AGA TAC GCG CA-3′, and AS, 5′-TGT GTG CGT GTA GGG GGT TA-3′ (Supplementary Fig. 4). PCR was performed with 12.5 µl of SmartGene 2 × pfu Mixed Taq Advanced (SJ Bioscience, Republic of Korea), 9.5 µl of nuclease-free water, 1 µl of primers (10 pmol/µl), and 1 µl of pGL4.11 vector cloned with V2 original construct as template DNA. The PCR conditions were as follows: initialization at 94 °C for 3 min, 22 thermal cycles of 94 °C for 40 s, primer-specific annealing at 61 °C for 30 s, 72 °C for 6 min, and a final elongation step at 72 °C for 5 min.

All PCR products were separated on a 1.5% agarose gel and purified with Expin Gel SV (GeneAll, Republic of Korea). The purified PCR products were cloned into a pGL4.11-T vector (Promega, USA) and ligated by LigaFast Rapid DNA Ligation System (Promega, USA). Plasmid isolation was performed with the Exprep Plasmid SV mini (GeneAll, Republic of Korea). The vector into which the PCR products were inserted was verified by colony PCR.

### Luciferase reporter assay

The HepG2 cells (human liver cancer cell) were cultured in Dulbecco’s modified Eagle’s medium (DMEM) supplemented with 10% (v/v) heat-inactivated fetal bovine serum (Gibco, USA) and 1% (v/v) antibiotics-antimycotic solution (Gibco, USA) at 37 °C in a 5% (v/v) CO_2_ incubator. HepG2 cells were purchased from the Korean Cell Line Bank (KCLB, Korea). Cells were plated in 24-well plates at 2 × 10^4^ cells/well density and grown to 70% confluence. Cells were transfected with 500 ng of the pGL4.11-PTGR1 plasmid (V1 original, V1 deletion, V2 original, and V2 deletion) or the pGL4.11 basic vector linked to luciferase (Promega, USA) using the Lipofectamine 2000 (Invitrogen, USA), as described in the manufacturer’s protocol. Additionally, miR-negative control (NC) composed of scrambled miRNAs, miR-6867 mimic (5′-UGU GUG UGU AGA GGA AGA AGG GA-3′), or miR-6867-5p inhibitor (5′-UGU GUG UGU AGA GGA AGA AGG GA-3′) (25 nM final concentration) (BIONEER, Korea) was co-transfected with the pGL4.11-V2 original plasmids. The miR-NC is composed of scrambled miRNAs. In addition, 100 ng of pRL-TK plasmid vector was used to normalize for transfection efficiency. After 24 h of transfection, the cells were washed with Dulbecco’s phosphate-buffered saline (DPBS) and lysed in luciferase lysis buffer. The activity of firefly luciferase and Renilla luciferase in the cellular extracts was measured using the dual-luciferase reporter assay system with a luminometer (Promega, USA). The relative luciferase activity was obtained by normalizing the firefly luciferase activity with Renilla luciferase activity. All assays were performed in triplicates.

### Cell transfection and reverse transcription-quantitative PCR (qRT-PCR)

HepG2 cells were seeded in a 6-well plate (1.7 × 10^6^ cells per well) for 24 h, and miR-NC, miR-6867 mimic, and miR-6867-5p inhibitor (25 nM final concentration) were transfected into each well using the Lipofectamine2000 reagent (Promega, USA), according to the manufacturer’s protocol.

Total RNA was isolated using the RiboEx™ of GeneAll RNA extraction kit (GeneAll, Republic of Korea), according to the manufacturer’s instructions. For the cDNA synthesis of mRNA or miRNA, the PrimeScript™ RT reagent kit (TaKaRa, Japan) and the HB miR Multi Assay Kit system II (HeimBiotek, Korea) were used.

To evaluate mRNA expression, quantitative real-time polymerase chain reaction (qRT-PCR) was performed on a QuantStudio 1 Real-Time PCR system (Thermo Fisher Scientific, USA) using SYBR Green qPCR Master Mix—Low ROX (Smart Gene, Republic of Korea). For miRNA expression, the HB miR Multi Assay Kit system I (HeimBiotek, Korea) was used to perform qRT-PCR. The target mRNA and miRNA expression levels were normalized using GAPDH and U6 as internal controls, respectively. All samples were analyzed in triplicate, and the relative expression values were determined using the 2^−ΔΔCt^ method^53^. The primers used for qRT-PCR were as follows; PTGR1 S, 5′-CAA CAA CAA CCA GTC ACC TCA-3′; PTGR1 AS, 5′-CCC TCC CTA TGT CCA TGT GT-3′; GAPDH S, 5′- GAA ATC CCA TCA CCA TCT TCC AGG-3′; GAPDH AS, 5′-GAG CCC CAG CCT TCT CCA TG-3′.

### Human RNA samples

Total RNA from normal human tissues (liver, kidney, fetal liver, small intestine, prostate, stomach, lung, skeletal muscle, uterus, and colon) was purchased from BD Bioscience Clontech.

### Statistical analysis

Experiments were performed at least three times, and the results were presented as mean ± standard deviation (SD). Statistical analyses of all data were conducted using MS Excel tools. The significance of differences between groups was analyzed using Student’s *t* test or one-way analysis of variance (ANOVA). Values of *p* < 0.05 were considered statistically significant (*p < 0.05, **p < 0.01).

### Supplementary Information

Supplementary Information 1.

Supplementary Information 2.

Supplementary Information 3.

Supplementary Information 4.

## Data Availability

The data underlying this article are available in the article and in its online supplementary material.

## References

[1] Ravichandran S, Subramani VK, Kim KK (2019). Z-DNA in the genome: From structure to disease. Biophys. Rev..

[2] Wang AH-J (1979). Molecular structure of a left-handed double helical DNA fragment at atomic resolution. Nature.

[3] Oh D-B, Kim Y-G, Rich A (2002). Z-DNA-binding proteins can act as potent effectors of gene expression in vivo. Proc. Natl. Acad. Sci..

[4] Rich A, Nordheim A, Wang AH-J (1984). The chemistry and biology of left-handed Z-DNA. Ann. Rev. Biochem..

[5] Peck LJ, Nordheim A, Rich A, Wang JC (1982). Flipping of cloned d (pCpG) nd (pCpG) n DNA sequences from right-to left-handed helical structure by salt, Co (III), or negative supercoiling. Proc. Natl. Acad. Sci..

[6] Rich A, Zhang S (2003). Z-DNA: The long road to biological function. Nat. Rev. Genet..

[7] Liu LF, Wang JC (1987). Supercoiling of the DNA template during transcription. Proc. Natl. Acad. Sci..

[8] Ellison MJ (1986). An assessment of the Z-DNA forming potential of alternating dA-dT stretches in supercoiled plasmids. Biochemistry.

[9] Schroth GP, Chou P-J, Ho PS (1992). Mapping Z-DNA in the human genome. Computer-aided mapping reveals a nonrandom distribution of potential Z-DNA-forming sequences in human genes. J. Biol. Chem..

[10] Shin S-I (2016). Z-DNA-forming sites identified by ChIP-Seq are associated with actively transcribed regions in the human genome. DNA Res..

[11] Li H (2009). Human genomic Z-DNA segments probed by the Zα domain of ADAR1. Nucleic Acids Res..

[12] Beknazarov N, Jin S, Poptsova M (2020). Deep learning approach for predicting functional Z-DNA regions using omics data. Sci. Rep..

[13] Maruyama A, Mimura J, Harada N, Itoh K (2013). Nrf2 activation is associated with Z-DNA formation in the human HO-1 promoter. Nucleic Acids Res..

[14] Lee D (2022). Z-DNA-containing long terminal repeats of human endogenous retrovirus families provide alternative promoters for human functional genes. Mol. Cells.

[15] Herbert A (2019). Z-DNA and Z-RNA in human disease. Commun. Biol..

[16] Olsen, U. D. J. G. I. H. T. B. E. P. P. R. P. W. S. S. T. D. N. C. J.-F. *et al.* Initial sequencing and analysis of the human genome. *Nature* **409**, 860–921 (2001). 10.1038/35057062 11237011

[17] Kazazian HH (2004). Mobile elements: Drivers of genome evolution. Science.

[18] Ayarpadikannan S, Kim H-S (2014). The impact of transposable elements in genome evolution and genetic instability and their implications in various diseases. Genom. Inform..

[19] Bennetzen JL (2000). Transposable element contributions to plant gene and genome evolution. Plant Mol. Biol..

[20] Lee H-E, Ayarpadikannan S, Kim H-S (2015). Role of transposable elements in genomic rearrangement, evolution, gene regulation and epigenetics in primates. Genes Genet. Syst..

[21] Abascal F, Tress ML, Valencia A (2015). Alternative splicing and co-option of transposable elements: The case of TMPO/LAP2α and ZNF451 in mammals. Bioinformatics.

[22] Esteller M (2011). Non-coding RNAs in human disease. Nat. Rev. Genet..

[23] Michlewski G, Cáceres JF (2019). Post-transcriptional control of miRNA biogenesis. Rna.

[24] Pu M (2019). Regulatory network of miRNA on its target: Coordination between transcriptional and post-transcriptional regulation of gene expression. Cell. Mol. Life Sci..

[25] Herbert A, Pavlov F, Konovalov D, Poptsova M (2023). Conserved miRNAs and flipons shape gene expression during development by altering promoter conformations. Int. J. Mol. Sci..

[26] Kurth R, Bannert N (2010). Beneficial and detrimental effects of human endogenous retroviruses. Int. J. Cancer.

[27] Bao W, Kojima KK, Kohany O (2015). Repbase Update, a database of repetitive elements in eukaryotic genomes. Mobile DNA.

[28] Zhang M (2015). Nrf2 is a potential prognostic marker and promotes proliferation and invasion in human hepatocellular carcinoma. BMC Cancer.

[29] Vongsutilers V, Gannett PM (2018). C8-Guanine modifications: Effect on Z-DNA formation and its role in cancer. Organ. Biomol. Chem..

[30] Zhang J (2006). BRG1 interacts with Nrf2 to selectively mediate HO-1 induction in response to oxidative stress. Mol. Cell. Biol..

[31] Saffer JD, Lerman MI (1983). Unusual class of Alu sequences containing a potential Z-DNA segment. Mol. Cell. Biol..

[32] Bourque G (2018). Ten things you should know about transposable elements. Genome Biol..

[33] Ayarpadikannan S, Lee H-E, Han K, Kim H-S (2015). Transposable element-driven transcript diversification and its relevance to genetic disorders. Gene.

[34] Grundy EE, Diab N, Chiappinelli KB (2022). Transposable element regulation and expression in cancer. FEBS J..

[35] Slotkin RK, Martienssen R (2007). Transposable elements and the epigenetic regulation of the genome. Nat. Rev. Genet..

[36] Hirsch CD, Springer NM (2017). Transposable element influences on gene expression in plants. Biochimica Biophys. Acta (BBA) Gene Regul. Mech..

[37] Wang X (2021). Prostaglandin reductase 1 as a potential therapeutic target for cancer therapy. Front. Pharmacol..

[38] Tobin DM, Roca FJ, Ray JP, Ko DC, Ramakrishnan L (2013). An enzyme that inactivates the inflammatory mediator leukotriene b4 restricts mycobacterial infection. PloS One.

[39] Vitturi DA (2013). Modulation of nitro-fatty acid signaling: Prostaglandin reductase-1 is a nitroalkene reductase. J. Biol. Chem..

[40] Kang G-J (2017). Novel involvement of miR-522-3p in high-mobility group box 1-induced prostaglandin reductase 1 expression and reduction of phagocytosis. Biochim. Biophys. Acta (BBA) Mol. Cell Res..

[41] Xue L (2016). Knockdown of prostaglandin reductase 1 (PTGR1) suppresses prostate cancer cell proliferation by inducing cell cycle arrest and apoptosis. BioScience Trends.

[42] Sánchez-Rodríguez R (2014). Increased expression of prostaglandin reductase 1 in hepatocellular carcinomas from clinical cases and experimental tumors in rats. Int. J. Biochem. Cell Biol..

[43] Dick RA, Kwak M-K, Sutter TR, Kensler TW (2001). Antioxidative function and substrate specificity of NAD (P) H-dependent alkenal/one oxidoreductase: A new role for leukotriene b412-hydroxydehydrogenase/15-oxoprostaglandin 13-reductase. J. Biol. Chem..

[44] Sánchez-Rodríguez R (2017). Ptgr1 expression is regulated by NRF2 in rat hepatocarcinogenesis and promotes cell proliferation and resistance to oxidative stress. Free Radic. Biol. Med..

[45] Dowell JA, Johnson JA (2013). Mechanisms of Nrf2 protection in astrocytes as identified by quantitative proteomics and siRNA screening. PloS One.

[46] MacLeod AK (2009). Characterization of the cancer chemopreventive NRF2-dependent gene battery in human keratinocytes: Demonstration that the KEAP1–NRF2 pathway, and not the BACH1–NRF2 pathway, controls cytoprotection against electrophiles as well as redox-cycling compounds. Carcinogenesis.

[47] Liu H, Mulholland N, Fu H, Zhao K (2006). Cooperative activity of BRG1 and Z-DNA formation in chromatin remodeling. Mol. Cell. Biol..

[48] Paugh SW (2016). MiRNAs form triplexes with double stranded DNA at sequence-specific binding sites: A eukaryotic mechanism via which miRNAs could directly alter gene expression. PLoS Comput. Biol..

[49] von Branderstein M (2018). Beyond the 3′ UTR binding–miRNA-induced protein truncation via DNA binding. Oncotarget.

[50] Ho PS, Ellison MJ, Quigley GJ, Rich A (1986). A computer aided thermodynamic approach for predicting the formation of Z-DNA in naturally occurring sequences. EMBO J..

[51] Paugh SW (2016). MiRNAs form triplexes with double stranded DNA at sequence-specific binding sites; a eukaryotic mechanism via which miRNAs could directly alter gene expression. PLoS Comput. Biol..

[52] Kent WJ (2002). The human genome browser at UCSC. Genome Res..

[53] Rao X, Huang X, Zhou Z, Lin X (2013). An improvement of the 2ˆ (–delta delta CT) method for quantitative real-time polymerase chain reaction data analysis. Biostat. Bioinform. Biomath..

