# Supplementary material for: The human PTGR1 gene expression is controlled by TE-derived Z-DNA forming sequence cooperating with miR-6867-5p
Source: Sci Rep. 2024 Feb 27;14:4723. doi: 10.1038/s41598-024-55332-x (PMC10899170; doi:10.1038/s41598-024-55332-x)

<Supplementary Figure Legend>

Supplementary Fig. 1. Structure of endogenous retrovirus (ERV). Internal portion (Int) includes gag, pro, pol, and env gene, excluding both LTRs. LTR, long terminal repeat; gag, group-specific antigen; pol, reverse transcriptase; pro, protease; env, envelope protein.

Supplementary Fig. 2. The entire sequence of MER4-int. Sequences shaded in blue indicate the region integrated into the *PTGR1* promoter.

Supplementary Fig. 3. Formation of TE-derived ZFS in *PTGR1* promoter. a ZHunt analysis of the entire sequence of MER4-int (1–6597bp). **b** Sequence alignment between the 1116–1160 bp sequence of MER4-int and MER4-int inserted into the *PTGR1* promoter.

Supplementary Fig. 4. Nucleotide sequences of *PTGR1* promoter. Thick arrows indicate primers used to design plasmid constructs. The square bracketed region indicates ZFS with two high z-score regions. To design the V2 deletion construct, the square bracketed region was deleted. The shaded gray rectangle indicates MER4-int element.

Supplementary Fig. 5. Relative expression of miR-6867-5p after transfection of miR-NC, miR mimic, and miR inhibitor. (**p<0.01)

Supplementary Fig. 6. Expression Analysis of NFE2L2 in several Liver cancer cell lines. The analysis was conducted The Human Protein Atlas. (https://www.proteinatlas.org/)

Supplementary Fig. 1

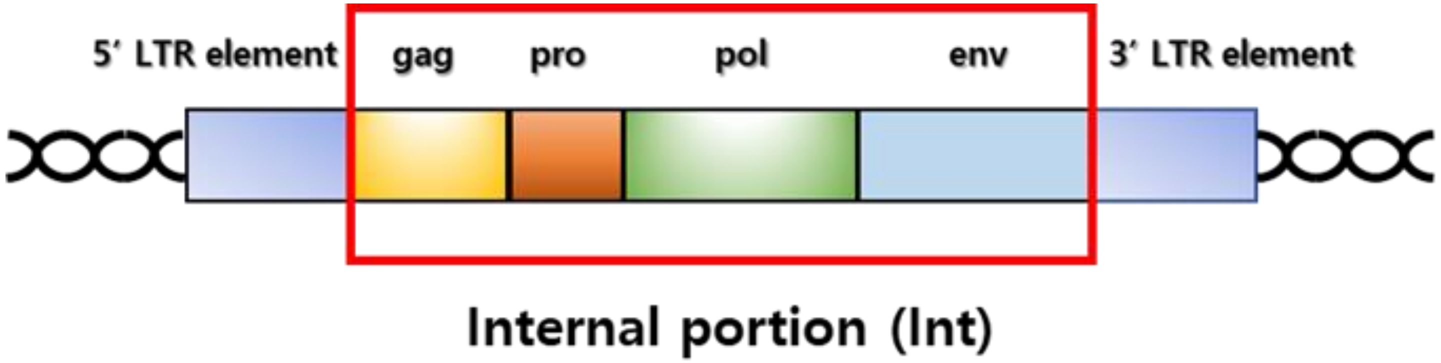

Supplementary Fig. 2

MER4I(MER4-int) (+) RepeatMasker v4.0.7 Dfam_2.0 : Current Datase

Accession numbers: DF0000806

1

TGGTAACCATGGAAGGGATTCTGAGTGGAGGTGCCCCTGACCTTTGACAAATCTCCTATTGGTGCTTGGTACCAGCATGAGCTAACTTTATGGCCCAAGCTTAAGATGTCTTTATGGCTCAAACCAATAGGACAATTTGCTGAGGTCTGGGAGCACCTCCCTCCAGAGAATCCCTGATCTCCCAAAATTTGGT
CGAGATCTAAAGTTTATTTTGCTGTACAACTCCTCTTTTTTTGGAGTTTTACTTGCTTCCAACACAAGGAAGGCAAGTTTTTCCTGCTTCCATGATGATGGAAGGCAGGTAACTCCTTTATGGAGTTTGAGCTCGCTTCCAACAGGGAAGATGAGTTTTTTTTCCTGCTTCTAGGATGGTAGAGAGCAGTCTTC
AGCCTGAGACCCATCCCTAGGTAAGTAACTGAATTGGGGTTTGTCTTGGAAATTCTCCTTCTTGGCTAAAAGTTAAGATTAACAACCTTGGAAATTCTCCTTAATGACTAAAAGTTAAGATTAACAACCAGCTGGTCTTAATTTCTCCTTACCATTAGAGCGCTCAGTAATCATATAAGTTGTGCGATCATTTGTTT
TGCTTAACTGTTTTTGTTTTTGTTGTTTTTGTTTGTTTCTGTTTTTGTTGTTGTTTCAGTCTTTTTCCCATTGGGTTTGACCAACTCTATCTGACTTGATCAATCTGGTGGGGGGAGAAAAATGGCCAGCAAAAGGAAAAAATTGAACTTGTTTAATTTGACTACTAAATCTGAAGGAAAATTCCAAATTATGGGGA
ACAAGGCCTCTGAAGTGGCTAAATTCCCACAAAAAAAAAAAAAAAAAAAAAAAAAAAAACAAGGCTAGCTTTTTGCTAGCCAGGCCAAACTGAAAGAGCAATGGCTGTCACCCCATGCTGCAGTTCCATAGCTAAGGTTCTGCCTTTTTTTTTTTTTTTCACCATGACAGCCTGGGTTTGGTTCCTAAATCAAGC
CCTTTCTGGTTTGATACTTGGTACTTCTGAAATAGCAGCAATTTGTCCTAGCTGAAATATGGTAATGAGATTTAAAAAGATTTTTTTAAAGGAGCTCAATGGTTAAAAGTCAGCTTAATTAAAAGGCTAACATCCAAGATGTGTGTGTGTATGTGTGCGTGTGTATGTGTGCATGTGTGTGTGTTTGTATTTAAAAGG
CCTTCATGTTTTTGTTTTGTTGTTTGTTTTTCTCTCCTAGGACCTTGTCTTTTTTTGAGCAAAGTTTTTTTCTTCTCAGTTGACTGAATTCTGTTTTCTTCATTTACTTCTGTTCGACCCTGTCTCTCCTTTCTCTTGCCACCTTCTGCTGCATGTGAAGGGACCTAAAATAGTTTTATAATAGCCTGGGGTTCCTTAAA
GAAAATGGAGAAAGGTGCCAGACACTCCCTTTTGGGGAGAAACTTCTGTTTTTCCTTATGGAACCCCAAGAGTAGTAAACAGACAAGTTCGTCTCAGCTCTTAAACTCTGCTTGCTTTTGTATTGTGTTACCTGATTTTTTGACTAAAATAGTTATTGCAACAGAGGCTACTCTTGGGTTTTTAAGGAAGAGTG
TAGTTTAGACACTTAGAAATGTCTTTGTTTAAAAATTTAAGTGCACTGTAAAAGCATCACATGGTCTAGCCTCATAATAATTCTCCCTTTTTGGAGACCCAGGATTCAGTGTGGGCTCTGCCCAGAGCTCAGAGATCCAGTTAAAATAGGTAGTCCCTATCTAAATAAAATTGGTCTCCTTATACAATCCTATGATA
GATTTCTATAATTTTATGTTTGATTTGGCATCAATTTTTAATCTCCCTCTAGCACCACCAGACTTTTTCTCTCTGTACCTTATGATGTAATTTTTGCTATTTGATTTTTCACCTAAGAGTTGTTTCCTTTAATATGCAAATTTAAGGCTATTTAGCTGACAACTGCCTAGGGTAGTGAAACAGGTTATCAAGAATTTGAA
AGTCTAAGATAGGAAAAAAAGGAGGTTTTATGAATCTATAAGATGTACTTCTATTGGCATGCCTAATACGTCTATGTATTTATGTGTTGTGTACACAATGTTTCACTACTAAAAATATATAAAAGAGCTCTAATTAATTGGCTTAAAGAAAAATAAAAGCACTTAAATCAAATACTTTATCAGGAAAAAAGAAAAGA
CTAGTCAAATGCTTTTTCAAGTTTATGAAATGCTTTTTCAAGTTTATGTAACTTAAGTAAAATCTTTAATAAATAAGCTAGCTTTAAAATTATTGGTAAAGTAATATTAGAAATGTCTTAAGAATTGCCAGCATACATTTTTGTTTGCATTTTATTAATCAAGCAATTTCATACTTATCCCTGCCAAATACTATAAGGGT
GTCAAAATTTGGCATAGGGGTTACAAAACTATAAACCCAGCCCAAAACAGAATGATCTTTGCTTGTGTAATTTTTAATAAATAAGACATTGATATTGGTTTAATGAAAATAGCTACATCTTGAATTTAGTAAGATTACCATAACTTTTAATCTTGTGGCTTTAGGCGATTTAGTCCATAGGCAGTAAAACTTATTGTT
TTACGAAATTACTTTTATAGTTGCATTTATGCTAAGCCATTGGCTGAGTCACTTAAAAAATTAGTTGTTGTCTCTGTTAAAGTTTAACAAAGATAGCTTAAAATTTAATTTTAAGTTTCCCTTACTTAGATCAAATCTTTTTTCACTGTCTCAGTTATAACATTGTAATGGTGGTTTAATAACTTTAAATGATGACTATCA
CAGTTTTCATAAATAATCTAGGTAAACAATTAATAAAATAATTAGGTAAATGTAATGGGATAAATACTTGTAGACAAACTTGTCATAATTTAGAATCTAAAGTTATATTAAATTAAATAATAGATATTTCATTATTTGGGTATTTTCCAATAAAAATATATTGTAGGAAAACATTCTTTCTTAAAAAAAAGTGTGTCCTTT
TTAAAAAAATGGTGAACAATTTTTGTCTAATTCAAAGCTTATTTAAAGGTCATGTATAAAACAAGGTAAAAGGAACCAGGAAATAAGAGAGATGTAAAGAAAGTTATAAAAATAAAGAGGTATTTTTTGTAGGTAAGAAAGCTTAAAGAGAAATAATTTTATATGAGAAAGAATCTTGTATGGTAAATTTATGA
GAAAGAATCTTGTATGGTAAATTTTTGTCCTAGAATAAAATGACTGGTTTTTAAGAAAGAGGATGTTCAGGACAAACCAGAAAGTCCAAGCATGTCATGAATGGTCTGTGTAAGTCACAATAAGATTTATATAAAAAAAAAATTAAAAAATTTTATATGATCAAGTTGTCTATAATTAAAGGGAAATTATTTATAATG
GTCTTTCTAGAGATTGGGGTTTGATGTAAAAAAAACACTTATACACTGAAAATTGGTTAGAACAATGAAATTTTCTTAAGGGATTGATTTACTCTTAAAATTATAAGAGATTTTAATTTTATCCCAAAGGTTCAACTTTTATTGCATCTCACTGTTTTCAGGTTTTCTCTCCCCTTTTAAAAGGCCTGAAATAGTAAC
TCTCTCCTTCAACTCATTTGTCAGCTCATGTAATTTTTTCCTCAGGTTCTAACTGTTGTTGTGGCCTGATGCTAAAAATGTTTTATCTTAAAGGTCTAAAGGAAATGTTTTCTTCCAACATAATATTCTGTGCTCTTCTTTTCTTGATATGTTTAAATTTTTCTATGAAACTAGAAAATTTCACTTATGACCCAGGACAC
ACTCTTCCTTGTCTAATTAATTCAAGTACCATTTTCATTAGTTTGACTTCAGGTTATCTAAAGGGCTCCCCATAGGGAAAAGCAGTCACACTGCAGAAGGTTTTTCTTTTGCCTTTTGGTAACTGGCCTAACAAACAGATTTTATGTTTTATTGAAATAATTCCTATGTCATTATTATTAAGTTTTTGGTTTGCTTAGG
AAAACTGAGATTAAAATTTTTTAAATTAAGGTTATTACATCCATGTATCTTTCTGTATTGCTTTTAAAGTCCTTGTGACATTGAGTTACAGGGCTTTGACTCCTGGGTCTAAAAAGGACACCAAGTCCTGCTAAATCTTAAACACTGACAGCAATTAAAGCCTCATCTTCAGGCCCGTAGAAGATGCCAATCAAA
ATAAAGTGCATTCCTGAGACACAGGAAATTAAAGCTATTCAACTCCTCAAGGCCCAGGGACTATCTGGAAGAGGTGGGCGTGTGAGATTGTAAGGGCCAATTTTGAGAGATAAAATAAGTTCAAAGTTTCTCTATAAATTAATCATTAAAAGTTTCTCTATAAATTAATCATTAATGTCAAAGGCACACTGATG
CAAGACCAGCATATGGGCCCCTGTGTCAGATTAACAAGGTTTTCTTGAAGCATTAACCAACTCCTTAATAAAGGTTATAAAGGTTATAAAAGGCTTATGGAAGTTATATCTTATGGTCAAGATTAAAATTTTATAGATTGTTTATAAAATTTTGAAAAACAAATTTAATTGGCTTCATGCTGTTTTTATTAGGGCTTA
TTGTTTGGAAAATTAAGTCTCCTCTCTCAAAGAATGAAGGTTTTTGCCTTTTTTGAAATCCTTGAGTTATCACTTTGGTTAAATGAATGACTTATTTTACAATGACCTGTGATCCTATTTTGTGATATCAAGTGTTTTAAACCTTTGATATTTGACAAACTTTCCAAAATCAAATTATAAATTATGTCTTTCTGACCTAA
TTAATCCTTTAAGATATTAGGTTCCCTAAAGTCCAAAAATGACATAATTTGGCTTATTTGGTATAAAAATTATACAGGAAGCATTGTCAAATATGAAAGGTGTTTGGTTTTCTTTGGGCTGTATTTGTATAAATATGTTATTGGTATGTGTTCCAAAATTATGGGAAACTCCTATAATTCTGATATGACTTAGTGTACATT
ATCAGTAATAATTATAATTGTTATGTTAAATTATTGTGTGCCACAGAGGTAACCAAATTTCCTTGTCAATTGTGTCTTTGACTATGGCTGTCCTAAAACTTTTGTCATCCATGGACAATTGTTTTGTTTTGGTCCTCTTTAGAAGGTGGTTTATAATCAGCTATAAAACTCTAACAGGTGCTCTTGAATGCAGGTTTCTG
ATAACTTTGGAGATTGTGACATCAGAATAGAGGAAAAACTTTCAGGACTTATGGAGAGCTGAAATGTTCATGAATATTGTTGATCCAATATCGAGCAAGCAGAACAGGAATTAACTGCATGGACTGAACTAATAGAAGACTGAAGTAATCTTTTTGACTTTTTGCTTAAAATGTTGCTGATCCTTTGTTTTGTTT
TTCAGAGTCAAGAAAACTTTTCTTTTGAGCTATTTACAGCTTTTAACAATTGAGTATAGTATACTCCTATGAACAAAATTTGGAGCATATTTGTTTCTCTCTACCTGATTTCTCCAGAATTTGGAAACTATTTGTGAGTATTCTTAACTTATGGCAATACAGTTATTTGCATAAGTGCAATAAGAATCTGTTTTCATTT
GTAACAGGACACAATTGGAGAAACTGGTTATTTTACCAAGGCTTTGACTGGAATGGTGTGCTTTCCTTTAAGGAATCAAACTTGACTTATGGAGCCAATAAAAGCCCTTGGGAAAACTGGCCTCATACCTTGTCTACACAGTCCCTGTACAGGGTTCCTGACCTGTGGTAAGTAAAGAATGTCACTTTCTGAC
AGGCCCAGGAGCCCCAAGTTTATCTTGGAACCTCAAGAGGAGAGGAAATTCACCCAACTCATAGGTATTTGATGGTACAAATCCATGGCTGGGCTTGGCTTTAAAAAAGTCGTATCTGAGATTCCTTCTATGGAACAAAGTTCCATCAAAGCCAATTTAAAAAGAGCCTATGTAAAAAATAATTATTCTTGCT
GCACTTTATACAAATAATCAGGCCAAGTATAATAAGACTAAAACTTATTTTGCAAGCAAATCAGTCCTACCATGATTTGTCTTTAGTAAAAATGGGAAACTGGAGAGAGAAAAATTATGTTTCAAAAACTATAGTACACCTGTTGTTAGATTCTAGTTGTTTTTCAATTTTTATTATTTTCTACAGTTTGGACTGAAT
TCTAAATTTTTTCTTGGCTACAAGTCTTCAAAATAATGTTTTCAATTTTTTTTCTTTTTTTCCTTCCTTTTTTCCCATTTTTCCTAATTTGGAGTCACTGAAAACTAAAGCTGTGCTTTTCTTAAAGCCCTGTGAACTGAAGCTAGACAACTTAAACTTCAGAAGAAAATAACAGCAACCTATTTACATACATAAGCC
ACTTTCATACCTGCCTACTGATGTATGGACTTCAGAGTAATGTGGCCTATATCAATTTTCCAGGATTGTTCTTTTGTTTGTTGTTGTTTTTCTCCCTTCCTCCCCCTATTTTCTCTTCATAGGACATGAGACTTCACAACCTGCTAAAAATGAGCTTTCCTAATAACTTGGGACCTACCCATCTAGGAATAAACCATCC
TAGCCATGAGAGATCAGATGAAACCTGAGGCCAGAGACTCATTTTCTTCTAAAATGCTTTCTCCAAAAGATTTTAAAAAAGAAGGGA 6597

: 1116-1160bp

Supplementary Fig. 3

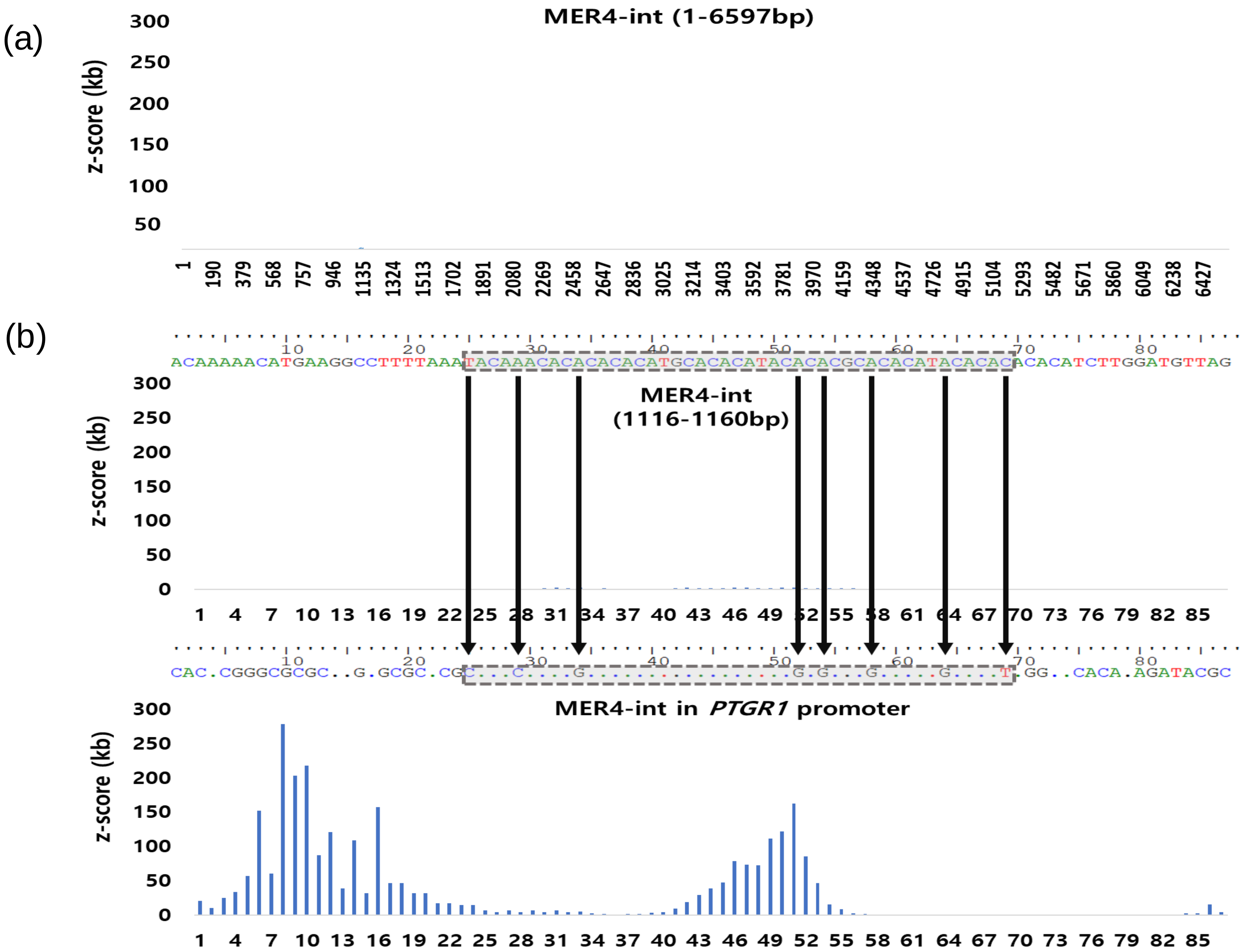

Supplementary Fig. 4

CTTGCAGATGGCTGAACTGAGTGAGGCAAGGAGAAGGTAAGTAAGTCTTTGAAAGCTACA 60

CCACTAGTAAGTGGCAGGTGCAGGCTTCGAACCTGCAGTCGTCTAATACCAACGACTTAG 120

CTTCACGTTCAACCCCTGCCTGAGACCACCTCTCCTTGCAGAAGCCAGGTCAAGGCCTGA 180

S1 primer

TGTTTGCTAGGGTGTGATGGGTGGGGTGCCTCAGTGGCAAGAGTGCTCACTCCTGCCTGG 240

GGGAGTCTGGCAAGGGTTCACAGAGGTGACCTTGAATTGGCTGGTGGAAGGCGAGTAGGT 300

GTTCAGCAGGCGGACTGGGGACAGACAGGCAGGGTGTCTGCACCGCAGCCCTGGGCGAAA 360

GCTTGCACTGCGGGCAAGCGGCAGGGCTGACGGGGAGCCCAGGGATCGGAGCCAGCACCG 420

GGGGCTTATTAGGGCTGCGGCCGCCCCAGGCCCTGCCTTTGGACCCTTCCGGCCTGCTTG 480

GCAGATGCCACGGTTGGTTCCCCCACGGGGCATATTTCCGCCGGGCTCCAACTTAGGACC 540

CCAGGCGGAGGTCCAGTGTCAGCTGGACAAAGCTCTGTTAGGAATTTTCCTATCAGGAGT 600

V1 TSS(+1)

CCCTAACCCCCTACACGCACACA[CACGGGCGCGCGGGCGCGCACGCACACACACGCACAC 660

AS1 primer

ATGCACACATACGCGCGCGCACATGCACATA]GGCACACAGAGATACGCGCACACACACAC 720

AS2 primer

ACAAACGCACTCAGATTTCCCGGACCCTGGTTTTCCTCCTGTGACCCTTTCGGGGCCGGG 780

CTCTCACCCTAAAGAAGCAGCCCCGCCCTGGGGTGGCCCCACCCTCTCTTGGGACCTGTC 840

V2 TSS(+210)

ATAAGTCGGACCCCGGGCGCCCGGCTGCGCAGTCCCAGCCGCCTTCCCCAGGCAGTGGAA 900

CCTTCGGGCTCCTGTAAGTAGACCTCCTGCTTTCTTTCCTTCCCTTTTCTCCCTCCTCTA 960

AS3 primer

ACCCCCTTGCTTTCTTCCTGCCAAGTTTCTAGACGCCCCAGTGGAGGAGGTTTGCTCTTA 1020

GACCTCCTCCCCTTACGTCGAAATGACCGCTGGGTCTTGCACTCGGAGGATCTTGGGAGC 1080

V3 TSS(+420)

CGACGTCCCGACGCCTCCCGCCCCCGCAGTTCCTTGGAGAGCTTGGAGCCGCGCGCCGGA 1140

GGGAATAGGAAAGCTTGGTTACAACCCGGGACACCCGGTAGCTTCCTGCACCCGGGAGGG 1200

GGCTGGCCAGGGAACTTGCTGGACTGCACAACTGGGGGAGGGTAGAAGAAGAAAGATCGG 1260

GGTGGGGATTCGGGAATGGGCAAGCGGGCGGCGGGCGGCGGGAGGCGAGGGTGGGAGCAA 1320

AGGGCGAGC

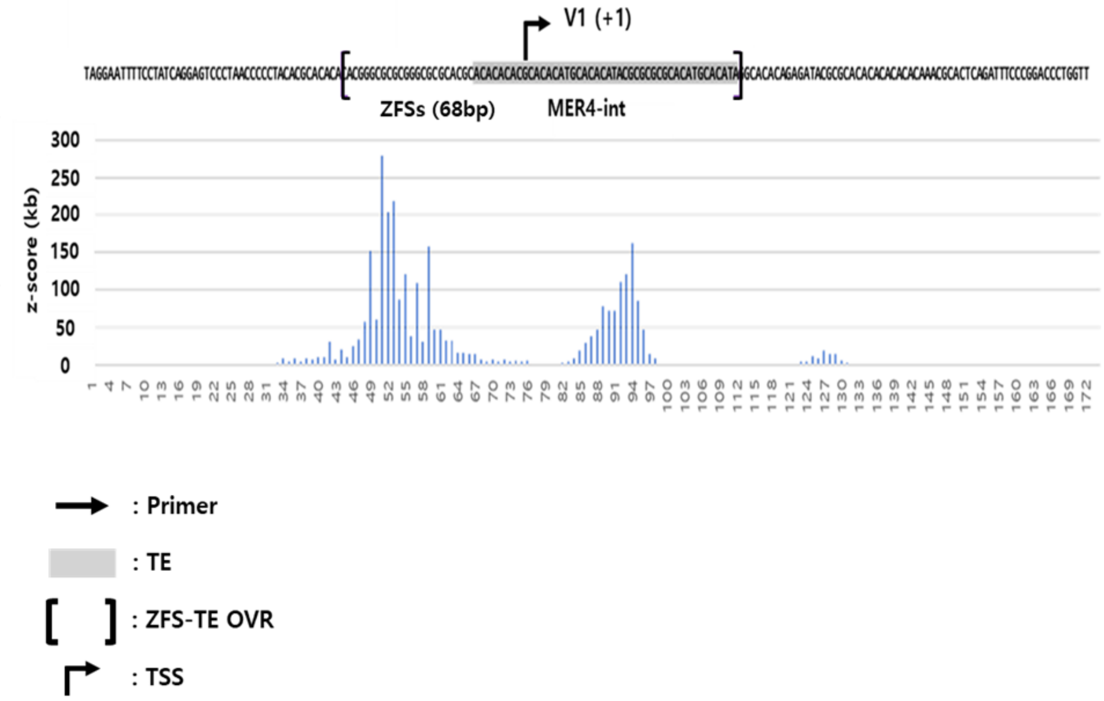

Supplementary Fig. 5

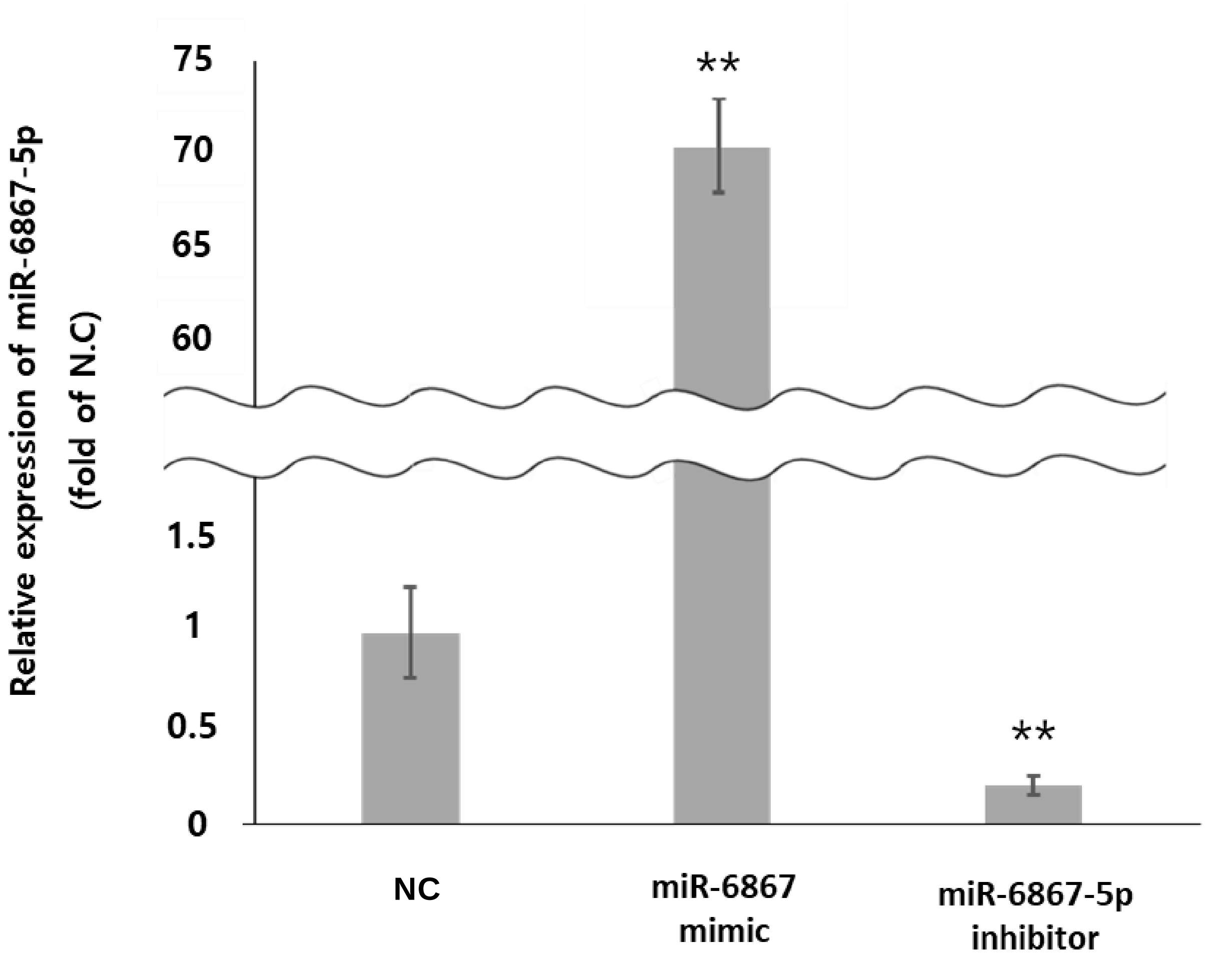

Supplementary Fig. 6

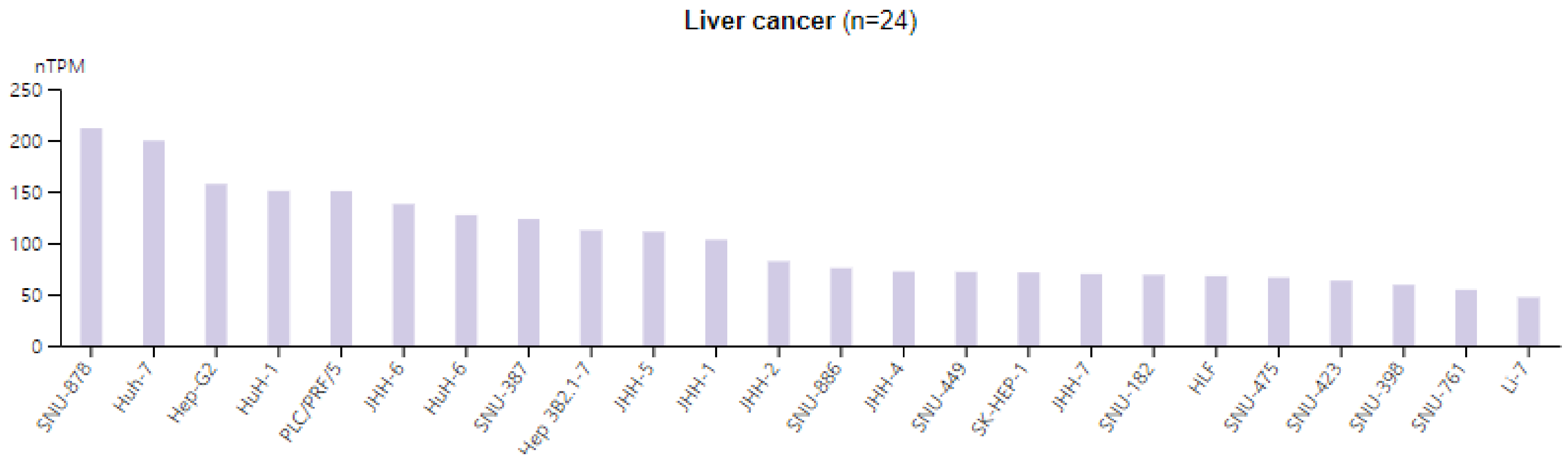

Supplement: Supplementary file 1 — Supplementary Information 1. [file 41598_2024_55332_MOESM1_ESM.pdf]
