# Supplementary material for: The human PTGR1 gene expression is controlled by TE-derived Z-DNA forming sequence cooperating with miR-6867-5p
Source: Sci Rep. 2024 Feb 27;14:4723. doi: 10.1038/s41598-024-55332-x (PMC10899170; doi:10.1038/s41598-024-55332-x)
Supplement: Supplementary file 2 — Supplementary Information 2. [file 41598_2024_55332_MOESM2_ESM.pdf]

Supplementary Fig. 7

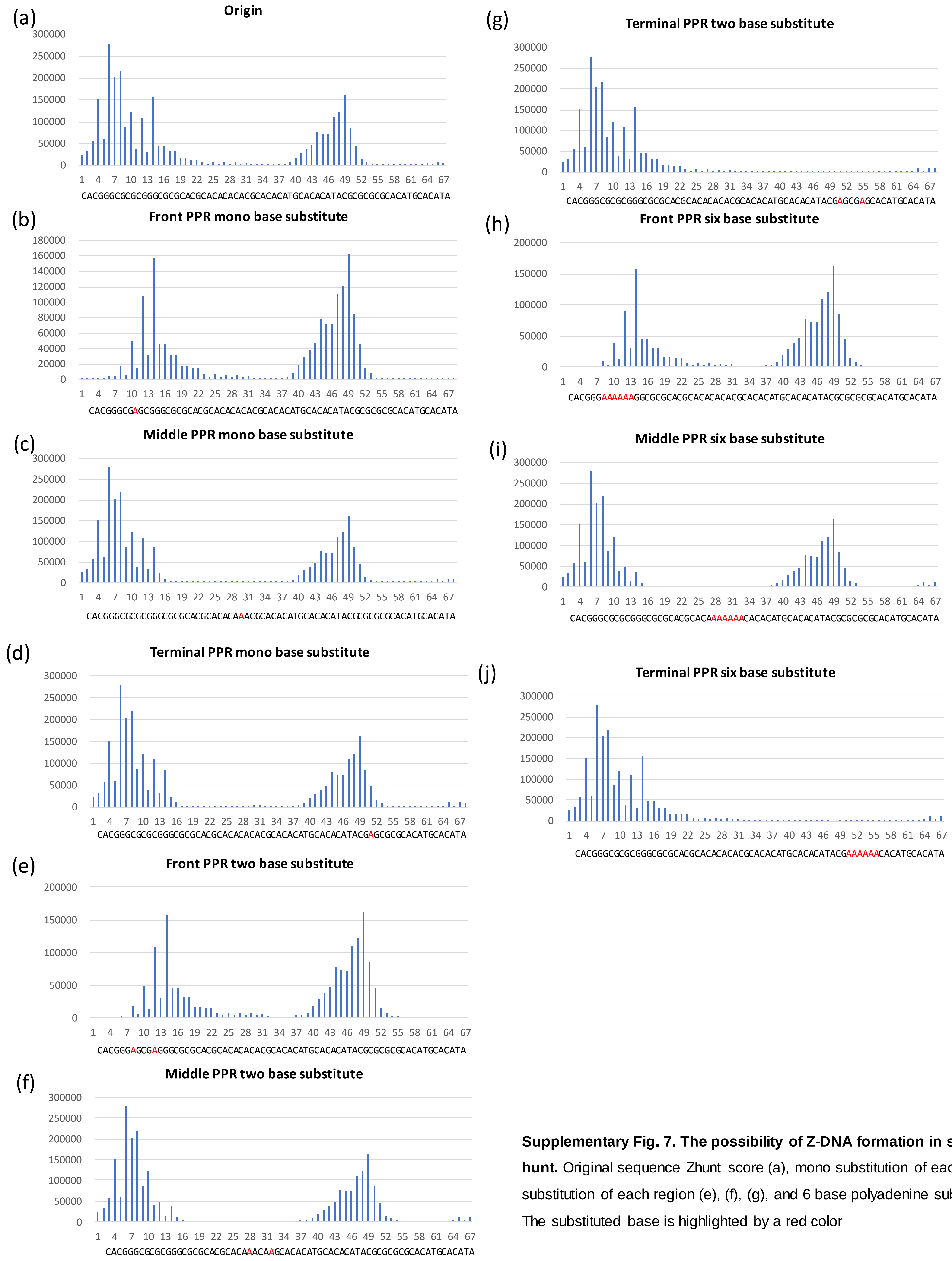

Supplementary Fig. 7. The possibility of Z-DNA formation in sequence mutation analyzed by Z-hunt. Original sequence Zhunt score (a), mono substitution of each region (b), (c), (d), two base substitution of each region (e), (f), (g), and 6 base polyadenine substitution of each region (h), (i), (j). The substituted base is highlighted by a red color
