# Supplementary material for: The human PTGR1 gene expression is controlled by TE-derived Z-DNA forming sequence cooperating with miR-6867-5p
Source: Sci Rep. 2024 Feb 27;14:4723. doi: 10.1038/s41598-024-55332-x (PMC10899170; doi:10.1038/s41598-024-55332-x)
Supplement: Supplementary file 3 — Supplementary Information 3. [file 41598_2024_55332_MOESM3_ESM.pdf]

Supplementary Fig. 8

(a)

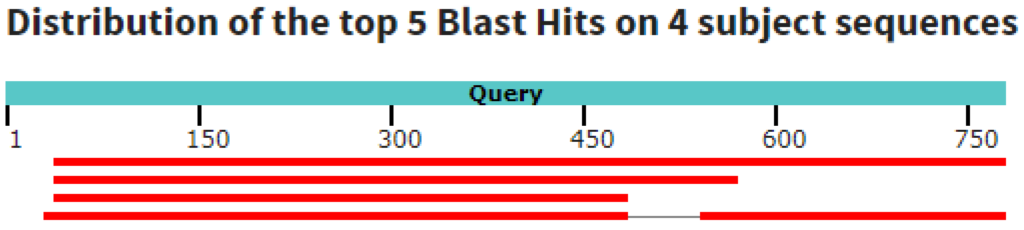

(b)

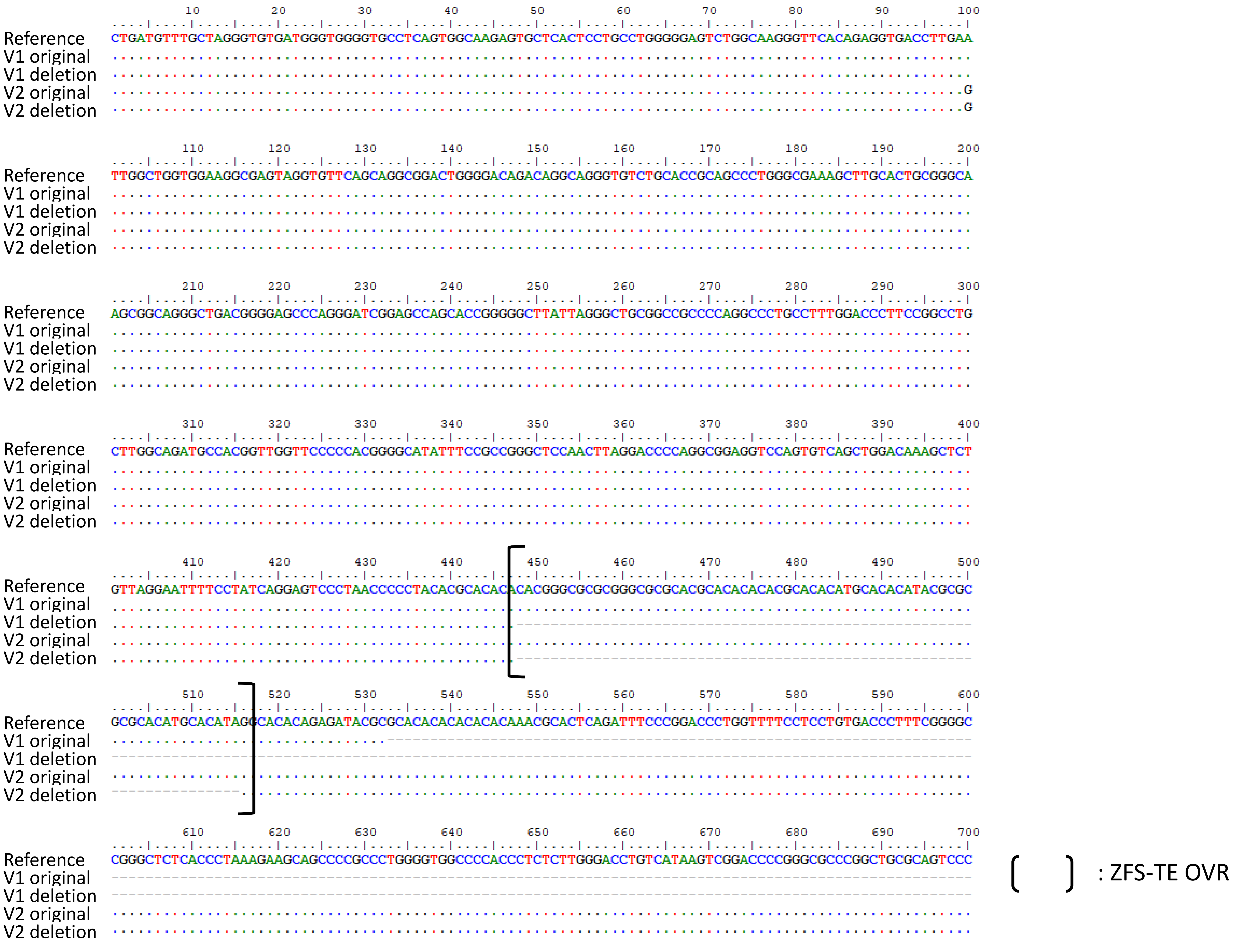

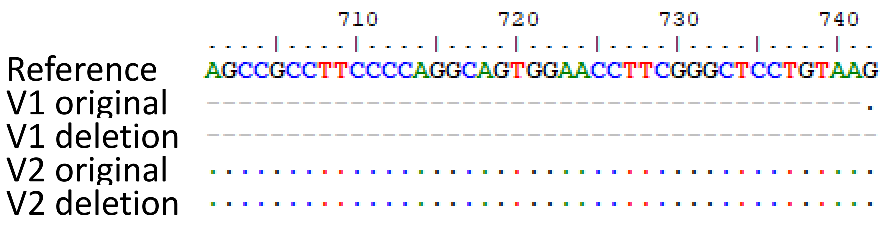

Supplementary Fig. 8. Sequence alignment of four PCR constructs and the hg19 reference sequence of the PTGR1 promoter region. Graphic summary of BLAST alignment (a) and alignment of sanger sequencing results of four PCR constructs with reference sequence (b).
